# Carnosine ameliorates postoperative cognitive dysfunction of aged rats by limiting astrocytes pyroptosis

**DOI:** 10.1016/j.neurot.2024.e00359

**Published:** 2024-04-25

**Authors:** Jiahong Shen, Jiawen Xu, Yuxin Wen, Zili Tang, Jiaqi Li, Jianliang Sun

**Affiliations:** a Department of Anesthesiology, Affiliated Hangzhou First People's Hospital, School of Medicine, Westlake University, Hangzhou, China; b Zhejiang University School of Medicine, Hangzhou, China; c Department of Anesthesiology, The Fourth Clinical School of Medicine, Zhejiang Chinese Medical University, Hangzhou, China

**Keywords:** Carnosine, Postoperative cognitive dysfunction, Astrocytes, Pyroptosis, NLRP3 inflammasome, Neuroinflammation

## Abstract

Postoperative cognitive dysfunction (POCD) is a common postoperative complication in elderly patients, and neuroinflammation is a key hallmark. Recent studies suggest that the NOD-like receptor family, pyrin domain containing 3 (NLRP3) inflammasome-mediated astrocytes pyroptosis is involved in the regulation of neuroinflammation in many neurocognitive diseases, while its role in POCD remains obscure. Carnosine is a natural endogenous dipeptide with anti-inflammatory and neuroprotective effects. To explore the effect of carnosine on POCD and its mechanism, we established a POCD model by exploratory laparotomy in 24-month-old male Sprague-Dawley rats. We found that the administrated of carnosine notably attenuated surgery-induced NLRP3 inflammasome activation and pyroptosis in astrocytes, central inflammation, and neuronal damage in the hippocampus of aged rats. In addition, carnosine dramatically ameliorated the learning and memory deficits of surgery-induced aged rats. Then in the in vitro experiments, we stimulated primary astrocytes with lipopolysaccharide (LPS) after carnosine pretreatment. The results also showed that the application of carnosine alleviated the activation of the NLRP3 inflammasome, pyroptosis, and inflammatory response in astrocytes stimulated by LPS. Taken together, these findings suggest that carnosine improves POCD in aged rats via inhibiting NLRP3-mediated astrocytes pyroptosis and neuroinflammation.

## Introduction

Postoperative cognitive dysfunction (POCD) is a common postoperative complication characterized by cognitive impairments associated with anesthesia and surgery, especially in elderly patients [1]. POCD has attracted increasing attention in recent years because it not only has a long-term impact on the quality of life following surgery but also has a huge burden on family and community healthcare [2]. However, little is known about the clinical treatment of POCD. Further exploration of its pathophysiological mechanism may provide a new approach to preventing and treating POCD.

Mounting evidence has shown that neuroinflammation plays an essential role in POCD [3]. Surgery activates the innate immune system resulting in the release of pro-inflammatory mediators, these processes negatively affect the blood-brain barrier (BBB), resulting in the infiltration of peripheral factors into the brain parenchyma [4]. The pro-inflammatory systemic milieu and neuroinflammation decrease synaptic plasticity and hippocampal neurogenesis, impairing learning and memory [5]. Furthermore, the cornu ammonis 1 (CA1) region of the hippocampus is highly vulnerable to pathological influences, the micro-structural alteration and consequent functional deterioration of critical hippocampal regions might result in clinical cognitive impairment [6]. Taking intervention to address central inflammatory response may be a neuroprotective strategy for POCD.

Astrocytes are the predominant glial cell type in the brain and perform key functions vital to central nervous system (CNS) physiology, including BBB formation and maintenance, synaptogenesis, neurotransmission, and metabolic regulation [7]. In addition, astrocytes play a critical role in neuroinflammation, they can promote neurodegeneration and inflammation through the release of pro-inflammatory cytokines and their intrinsic neurotoxic activities [8,9]. Therefore, inhibiting the excessive activation of astrocytes and subsequent inflammatory response holds great promise as a strategy to reduce surgery-induced brain damage.

Pyroptosis is an inflammatory type of regulated cell death, which occurs downstream of inflammasome activation [10]. Inflammasomes function as intracellular sensors of both microbial pathogens and foreign as well as host-derived danger signals that exist in CNS-resident cell types, including astrocytes, microglia, and neurons [11]. Inflammasome activation follows two pathways: a canonical inflammasome pathway activates caspase-1 through the ASC, and a noncanonical inflammasome activates caspase-4, 5, 11. Active caspase-1 and caspase-4/5/11 cleave gasdermin D (GSDMD) within the linker between its N-terminal and C-terminal. The released GSDMD-N domain binds to phosphoinositides in the plasma membrane and oligomerizes to form membrane pores. The pores disrupt the osmotic potential resulting in cell swelling and lysis and serve as a gate for the release of interleukin-1β (IL-1β) [12]. Growing studies have shown that pyroptosis plays an important role in neurocognitive diseases such as Alzheimer's disease (AD), Parkinson's disease (PD) [13,14], stroke [15], and perioperative neurocognitive disorders (PND) [16]. Furthermore, it has been found that a series of cell types in the CNS including astrocytes may undergo the NOD-like receptor family, pyrin domain containing 3 (NLRP3) inflammasome-induced pyroptosis [17]. The NLRP3 inflammasome consists of a sensor (NLRP3), an adaptor (ASC), and an effector (caspase-1) [18]. NLRP3 has high expression levels in astrocytes, the activation of which plays a predominant role in pyroptosis [19]. NLRP3 inflammasome activation and pyroptosis in astrocytes have been confirmed to exist in CNS diseases including sepsis [20] and depression [21]. However, it is not clear whether astrocyte pyroptosis is involved in the pathogenesis of POCD. Therefore, further investigation of the role of astrocyte pyroptosis in surgery-induced neurological injury is warranted.

Carnosine is a naturally occurring hydrophilic dipeptide composed of β-alanine and l-histidine, which is widely distributed in tissues including the brain and muscle [22]. The favorable toxicological properties of this natural molecule [23] and its ability to cross the BBB [24] make it a therapeutic potential for CNS diseases. Recently, carnosine has been shown to exert a multimodal activity including inhibition of protein cross-linking and aggregation of β-amyloid and related proteins, antioxidant, anti-inflammatory, and neuroprotective effects [25]. It could thus play an important role in the prevention and treatment of neurodegenerative diseases such as cerebral ischemia [26], AD [27], and PD [28]. It was reported that carnosine-induced neuroprotection in a model of subcortical ischemic vascular dementia (SIVD) is dependent on the suppression of astrocyte activation and inflammatory cytokine release [29]. However, so far, no research has focused on the role of carnosine in the regulation of inflammasome activation-dependent pyroptosis in the field of POCD. Accordingly, we hypothesized that carnosine might ameliorate the cognitive impairment caused by surgery by inhibiting astrocytes pyroptosis and neuroinflammation.

To investigate this hypothesis, we established an in vivo model of POCD induced by exploratory laparotomy and an in vitro model of typical inflammation stimulated by lipopolysaccharide (LPS) to explore the neuroprotective mechanism of carnosine.

## Materials and Methods

### Drugs and reagents

Carnosine and LPS (Escherichia coli O111:B4) were obtained from Sigma-Aldrich (St.Louis, MO, USA). Dulbecco's modified Eagle's medium (DMEM), 0.25% trypsin-Ethylenediaminetetraacetic acid (EDTA) solution, and fetal bovine serum (FBS) were purchased from Gibco-BRL (Grand Island, NY, USA). Phosphate-buffered saline (PBS), Rat IL-1β Immunoassay Kit, Rat tumour necrosis factor-α (TNF-α) Immunoassay Kit, Rat interleukin-6 (IL-6) Immunoassay Kit, Cell counting kit-8 (CCK-8), and primary antibodies for rabbit antibodies against NLRP3, ASC, IL-1β, Inducible nitric oxide synthase (INOS) were acquired from Boster (Wuhan, China). Mouse anti-glial fibrillary acidic protein (GFAP) monoclonal antibody was obtained from Cell Signaling Technology (Boston, MA, USA). Rabbit antibodies against GSDMD, GSDMD-N, Caspase-1, and Cleaved-Caspase-1 (Cl-Caspase-1) were purchased from Affinity Biosciences (Jiangsu, China). Secondary antibodies for horseradish peroxidase (HRP)-conjugated goat anti-rabbit, HRP-conjugated goat anti-mouse, Goat anti-mouse IgG conjugated CY3, and goat anti-rabbit IgG conjugated Alexa-Fluor 488 were from Boster. Bicinchoninic acid (BCA) kit, radioimmunoprecipitation (RIPA) buffer, and propidium iodide (PI) solution were acquired from Beyotime (Shanghai, China). Nissl staining kit, mouse anti-NeuN, and bovine serum albumin (BSA) were obtained from Servicebio (Wuhan, China).

### Animals and drug administration

**Fig. 1:**
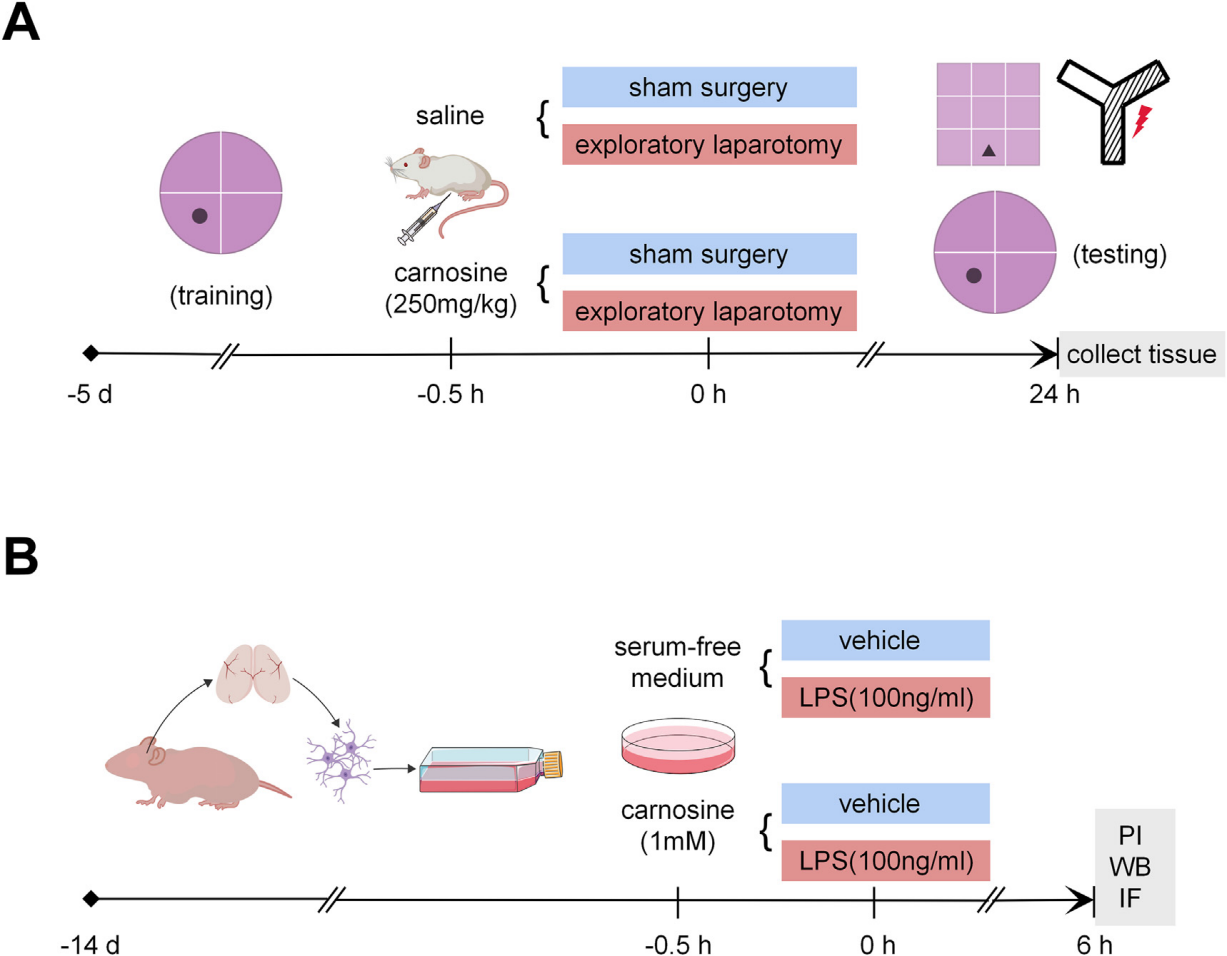
Outline of the experimental procedure. **A** In vivo experiment, aged rats were subjected to exploratory laparotomy. **B** In vitro, primary astrocytes from the brains of neonatal rats were used to detect the role of carnosine on postoperative cognitive dysfunction.

Aged (24-month-old) male Sprague-Dawley (SD) rats weighing 600–700 ​g were used in the study and obtained from Zhejiang Chinese Medical University Laboratory Animal Research Center. The cages of rats were under stable conditions with alternating light/dark cycles (12:12), environmental temperature 25 ​± ​1 ​°C, room humidity 50 ​± ​5%, and free access to food and water. All the experimental procedures were conducted following the Ethics Committee of Zhejiang Chinese Medicine University. For research, rats were randomly placed into four experimental groups. Sham: rats treated with saline intraperitoneally and sham operation; Surgery (Sur): rats treated with saline intraperitoneally and exploratory laparotomy; Carnosine (Car): rats treated with 250 ​mg/kg carnosine intraperitoneally and sham operation; Sur ​+ ​Car: rats treated with 250 ​mg/kg carnosine intraperitoneally and exploratory laparotomy (Fig. 1 A). Carnosine was dissolved in 0.9% sterile saline. All rats received intraperitoneal injections of carnosine or equal volume saline half an hour before surgery. The dose of carnosine was chosen based on previous evidence in which carnosine had a neuroprotective effect by alleviating apoptosis and oxidative stress in the prefrontal cortex and hippocampus regions of rats [30]. Behavioral tests and brain tissue collection were performed 24 ​h after the operation. The experimenters who carried out behavioral tests and sample tests kept blind to the grouping.

### POCD model

To establish a rat model of POCD, an aseptic exploratory laparotomy was performed under sevoflurane anesthesia (7–8% for induction and 3–4% for maintenance) as previously reported [31,32]. The abdominal region of the rat was shaved, and sterilized with iodophor and ethanol. Next, the abdominal cavity was exposed by a vertical incision of 3 ​cm at about 0.5 ​cm below the right lower rib of the rat. The surgeon used sterile tweezers to explore the viscera, intestines, and musculature in turn, then took out about 10 ​cm of the intestine and rubbed between the thumb and index finger for 30 ​s. After the intestine was put back into the peritoneal cavity, the incision was sutured from the peritoneal muscles to the skin with sterile 4-0 sutures and disinfected with iodophor. 0.25% ropivacaine infiltrated the incision to relieve surgery-related pain. The rat was placed on a heat blanket until anesthesia recovery. Rats in the sham group were anesthetized, shaved, and disinfected according to the above methods. The duration of anesthesia was the same as that in the surgery group, about 25 ​min.

### Enzyme-linked immunosorbent assay (ELISA)

Protein samples were extracted from the supernatant of rat hippocampal tissue and quantitatively analyzed by BCA kit. The levels of cytokines (IL-1β, TNF-α and IL-6) were detected with ELISA kits according to the manufacturer's instructions. The absorbance was measured at a wavelength of 450 ​nm using a microplate reader. Concentrations were calculated using ELISACalc software, expressed as a picogram per millilitre.

### Nissl staining

The coronal paraffin sections of 5 ​μm were routinely dewaxed to water. After three times of wash, the sections were stained with Nissl staining solution for 5 ​min at 37 ​°C, then dehydrated with alcohol and sealed. The slides were observed under a digital pathological section scanning analyzer (OLYMPUS, Japan).

### Open field test (OFT)

OFT was performed at 24 ​h post-surgery to test whether the impairment of learning ability induced by surgery was caused by the change of spontaneous motor ability after the operation. OFT was conducted as previously described with minor modifications [33]. The OFT was carried out in a grey opaque plastic chamber (100 ​cm ​× ​100 ​cm ​× ​40 ​cm). After a 5 ​min acclimation, each rat was placed in the center of the field and allowed to explore for 5 ​min. Before the next rat started, we removed the smell from the arena with alcohol and dried the chambers completely. The video tracking system automatically recorded the trajectory, traveling distance, and duration in the open field.

### Y maze test

The Y maze is a symmetrical three-arm maze. The Y maze was conducted according to the previous report, with slight modifications [34]. There was a lamp at the end of each arm. The lighted arm was a safe area, while the other arms and central area had 30 ​V electrical stimulation. After 3 ​min of adaptation, each rat began the test, with the illuminated arm (safe area) as a new starting area. We changed the safe area using a randomization method. If the rat reached the safe area within 10 ​s, this test was considered successful. We waited for the rat to reach the illuminated arm before the next stimulation. If nine of ten consecutive stimuli responded correctly, the rat was defined as meeting the learning criteria. The total number of stimuli during the training period was recorded as learning ability.

### Morris water maze (MWM) test

The MWM test is a behavioral experimental method that is mainly used to detect the spatial memory ability of rodents. The MWM test was performed as previously reported, with minor modifications [35]. The MWM encompassed a circular container with a diameter of 210 ​cm and a height of 51 ​cm, which was filled with water at a temperature of 19–22 ​°C. The pool was divided into four sectors. A target platform was located in a target quadrant (SW), and the water surface was about 1 ​cm higher than the target platform. Five-day training trials were conducted before surgery, all rats were trained three times a day. During the training days before the operation, the rats were gently put into the water from different quadrants to swim until they found the hidden platform. If rats failed to reach the platform within 90 ​s, they would be guided to the platform. In the end, rats stayed on the platform for 10 ​s, then they were dried and placed in a warm cage. The probe trial was carried out 24 ​h after surgery, the platform was removed. We selected the SW quadrant as the entry quadrant and put rats into the water to swim freely for 90 ​s. Using a video tracking system to record swimming speed, times of platform crossing and time spent in the target quadrant to evaluate the spatial memory ability of rats.

### Cell culture and treatment

Rat primary astrocytes were prepared according to the previous protocol, with slight modifications [36]. First, whole brains were isolated from neonatal SD rats, and the meninges and blood vessels were completely removed in cold PBS. Then, the tissues were minced with sterile ophthalmic scissors and digested with 0.25% trypsin-EDTA for 10 ​min at 37 ​°C. The digestion was terminated by adding a high-glucose DMEM medium containing 10% FBS equal to the volume of trypsin solution. The dissociated cells were passed through a 100 ​μm cell strainer, pelleted at 1500 ​rpm for 5 ​min, and re-suspended in a culture medium. The cells were seeded in cell culture flasks pre-coated with poly-d-lysine and cultured in a humidified atmosphere of 37 ​°C, 5% CO2 and 95% air. The culture medium was replaced every 3 days after seeding. When the glial cells formed a confluent monolayer (10–14 days), the astrocytes were separated from the microglia by shaking. The cultures were passaged into new 10-cm petri dishes at least three times at intervals of two weeks to obtain high-purity astrocytes culture. More than 95% of the cells were astrocytes confirmed by GFAP immunostaining. Carnosine was dissolved in a serum-free medium. Astrocytes were treated with different concentrations of carnosine for 6 ​h or pretreated with different concentrations of carnosine for 0.5 ​h and then stimulated with LPS (100 ​ng/ml) for 6 ​h. After selecting the most appropriate concentration of carnosine, four groups were designed for the next experiment. Control (Con): cultured in serum-free medium for 6 ​h; LPS: cultured in serum-free medium containing 100 ​ng/ml LPS for 6 ​h; Car: cultured in serum-free medium containing 1 ​mM carnosine for 6 ​h; LPS ​+ ​Car: pretreated with 1 ​mM carnosine for 0.5 ​h and then stimulated with 100 ​ng/ml LPS for 6 ​h (Fig. 1 B).

### Cell counting kit-8 cell viability assay

The viability of primary astrocytes was assessed by CCK-8 according to the instructions. Astrocytes were seeded in 96-well plates at a density of 5 ​× ​10^3^ ​cells per well and treated as designated. Then, the culture medium was replaced with 100 ​μl fresh serum-free medium containing 10 ​μl CCK-8 reagent, and the plates were incubated for 2 ​h at 37 ​°C in a humidified incubator with 5% CO2. The optical density (OD) of each well was measured at a wavelength of 450 ​nm using a microplate reader.

### Propidium iodide staining

Based on the previous report [35], we used PI staining to confirm the formation of the membrane pores in astrocytes. Astrocytes were seeded into 6-well plates. After treatment, the cells were washed with PBS and then stained with a PI solution (100 ​μg/ml) for 15 ​min at 37 ​°C in the dark. PI-positive cells with red fluorescence and cell morphology were photographed with a Zeiss fluorescence inverted microscope. The number of PI-positive cells was counted manually, and the data were expressed as a proportion of total cells.

### Western blotting

Hippocampal tissues and astrocytes were lysed in a clod RIPA buffer containing protease inhibitors and phosphatase inhibitors. The lysate was centrifuged and the concentration of the extracted supernatant was measured using a BCA kit. Proteins in extracts were denatured with sodium dodecyl sulfate (SDS) sample buffer and separated by 10% or 12% SDS-polyacrylamide gel electrophoresis. After electrotransferred onto polyvinylidene fluoride (PVDF) membranes, the bands were sealed with 5% BSA for 1 ​h at room temperature and then incubated with different primary antibodies overnight at 4 ​°C. The following primary antibodies were used: anti-NLRP3 (1:1000), anti-ASC (1:1000), anti-Caspase-1 (1:1000), anti-Cl-Caspase-1 (1:1000), anti-INOS (1:1000), anti-GSDMD (1:1000), anti-GSDMD-N (1:1000), anti-IL-1β (1:1000) and anti-β-actin (1:5000). After incubating the membranes with HRP-conjugated secondary antibodies for 1 ​h at room temperature, the protein bands were detected by enhanced chemiluminescence reagent. Using Image J software (NIH, Bethesda, MD, USA) to obtain the relative density of protein bands.

### Immunofluorescence

Brains were harvested at 24 ​h after operation and immersed with 4% paraformaldehyde for 24 ​h. After paraffin embedding, the tissues were sectioned coronally with a thickness of 5 ​μm. The slices were dewaxed, hydrated, and blocked with 1% BSA for 30 ​min at 37 ​°C, then incubated with primary antibodies against GFAP together with NLRP3 or GSDMD-N at 4 ​°C overnight. Slices of NeuN staining were incubated with mouse anti-NeuN. Sections were washed in PBS and incubated with CY3 and Alexa-Fluor 488 secondary antibodies for 1 ​h at 37 ​°C in the dark. Nuclear staining was counterstained with DAPI. The fluorescent images were captured using Pannoramic MIDI, Pannoramic 250FLASH, and Pannoramic DESK (3DHISTECH, Hungary).

Astrocytes were seeded into fluorescent dishes. After treatment, cells were washed and fixed with 4% paraformaldehyde for 30 ​min and then washed with PBS three times. After permeating for 15 ​min with membrane breaking solution, cells were blocked with 3% BSA for 30 ​min, followed by incubated with primary antibodies against GFAP (1:300) together with NLRP3 (1:150) or GSDMD-N (1:150) at 4 ​°C overnight. After three washes with PBS, cells were incubated with CY3 (1:100) and Alexa-Fluor 488 (1:100) secondary antibodies for 1 ​h at 37 ​°C in the dark, and the nuclei were stained with DAPI for 5 ​min. The fluorescent images were obtained by a confocal microscope (Carl Zeiss, Germany).

### Statistical analysis

GraphPad Prism 9.5 statistical package was used to analyze the data. The values were presented as mean ​± ​SD. The normality of data was evaluated individually. Brown-Forsythe test was used to verify the homogeneity of variance. Differences between groups were determined by one-way or two-way analysis of variance (ANOVA) followed by Tukey's multiple comparisons test. A value of p ​< ​0.05 was regarded as statistically significant.

## Results

### Carnosine improved learning and memory deficits induced by surgery in aged rats

**Fig. 2:**
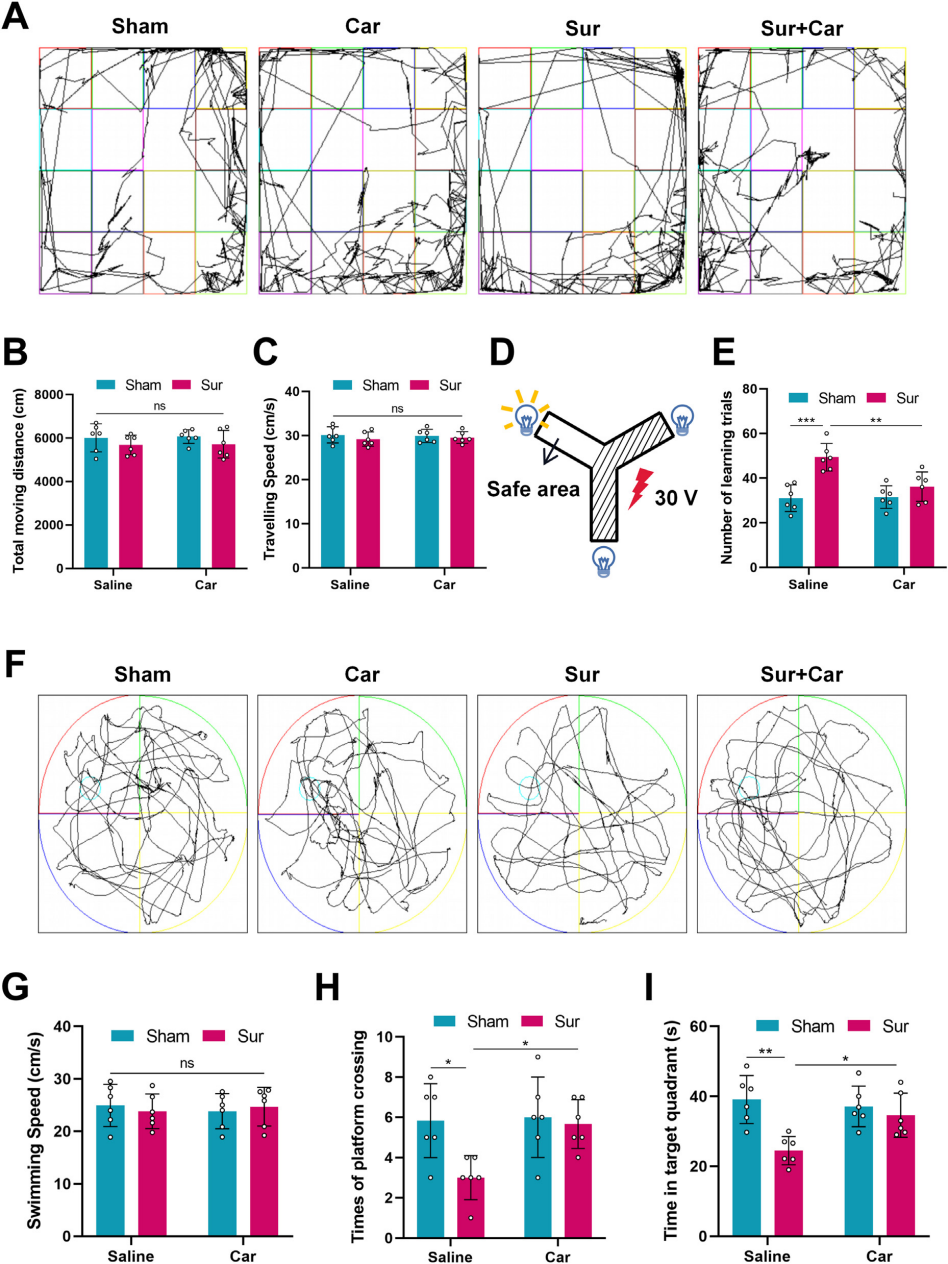
Effects of carnosine on surgery-induced learning and memory deficits in aged rats. Motor ability was detected by Open field test (OFT). **A** Trajectory maps during OFT at 24 ​h after surgery. **B** Total moving distance of OFT. **C** Traveling speed of OFT. Learning was assessed using Y maze test. **D** Schematic diagram of Y maze test. **E** Numbers of learning trials in Y maze (Sham: Sur, F (1, 20) ​= ​7.497, p ​= ​0.0002; Sur: Sur ​+ ​Car, F (1, 20) ​= ​5.384, p ​= ​0.0056). Memory was assessed using Morris water maze test (MWM). **F** Representative records of trajectory map of rats swimming in MWM. **G** Swimming speed. **H** Times of platform crossing (Sham: Sur, F (1, 20) ​= ​4.382, p ​= ​0.0267; Sur: Sur ​+ ​Car, F (1, 20) ​= ​4.124, p ​= ​0.0392). **I** Time in targeted quadrant during the testing phase (Sham: Sur, F (1, 20) ​= ​6.112, p ​= ​0.0017; Sur: Sur ​+ ​Car, F (1, 20) ​= ​4.243, p ​= ​0.0329). All the data are expressed as mean ​± ​SD (n ​= ​6 per group). ∗p ​< ​0.05, ∗∗p ​< ​0.01, ∗∗∗p ​< ​0.001.

Previous studies have shown that exploratory laparotomy could induce significant cognitive and memory decline after the operation [37,38]. Therefore, to verify our hypothesis, we first established an aged rat model of POCD induced by exploratory laparotomy to explore the effects of carnosine on brain protection. To investigate the effect of carnosine on POCD, we performed multiple behavioral tests including OFT, Y maze and MWM. First of all, We used OFT to observe the exercise ability of rats. The movement ability of rats in each group was not significantly affected 24 ​h after the operation (Fig. 2 A), and there was no difference in the total moving distance (Fig. 2 B) and traveling speed (Fig. 2C) of the rats in each group. On this basis, we carried out a Y maze to test the learning ability of rats. As shown in the figure, rats should enter the safe area within 10 ​s after the light is on, or they would be subjected to a 30 v shock (Fig. 2 D). The success of 9 out of 10 times was regarded as that rats had learned. The total number of times is the number of learning trials. Rats exposed to surgery showed a significant increase in the number of learning trials compared with the sham group. Nevertheless, this change was markedly reversed by the administration of carnosine, indicating that carnosine was effective in ameliorating surgery-evoked learning deficit (Fig. 2 E). Then, we employed MWM to evaluate the memory ability of rats. In the probe trial, there was no significant difference in swimming speed among the four groups (Fig. 2 G). We found that surgery markedly decreased the times of platform crossing and the time stay in the target quadrant. Meanwhile, carnosine treatment significantly improved the times of platform crossing than the surgery group (Fig. 2H). Carnosine administration also increased the time spent in the target quadrant (Fig. 2 I). These results indicated that carnosine could improve memory impairment caused by surgery. All these findings suggested that carnosine intraperitoneal treatment had a protective effect against surgery-induced learning and memory deficits in aged rats.

### Carnosine attenuated central inflammation and neuronal injury induced by surgery in the hippocampus of aged rats

**Fig. 3:**
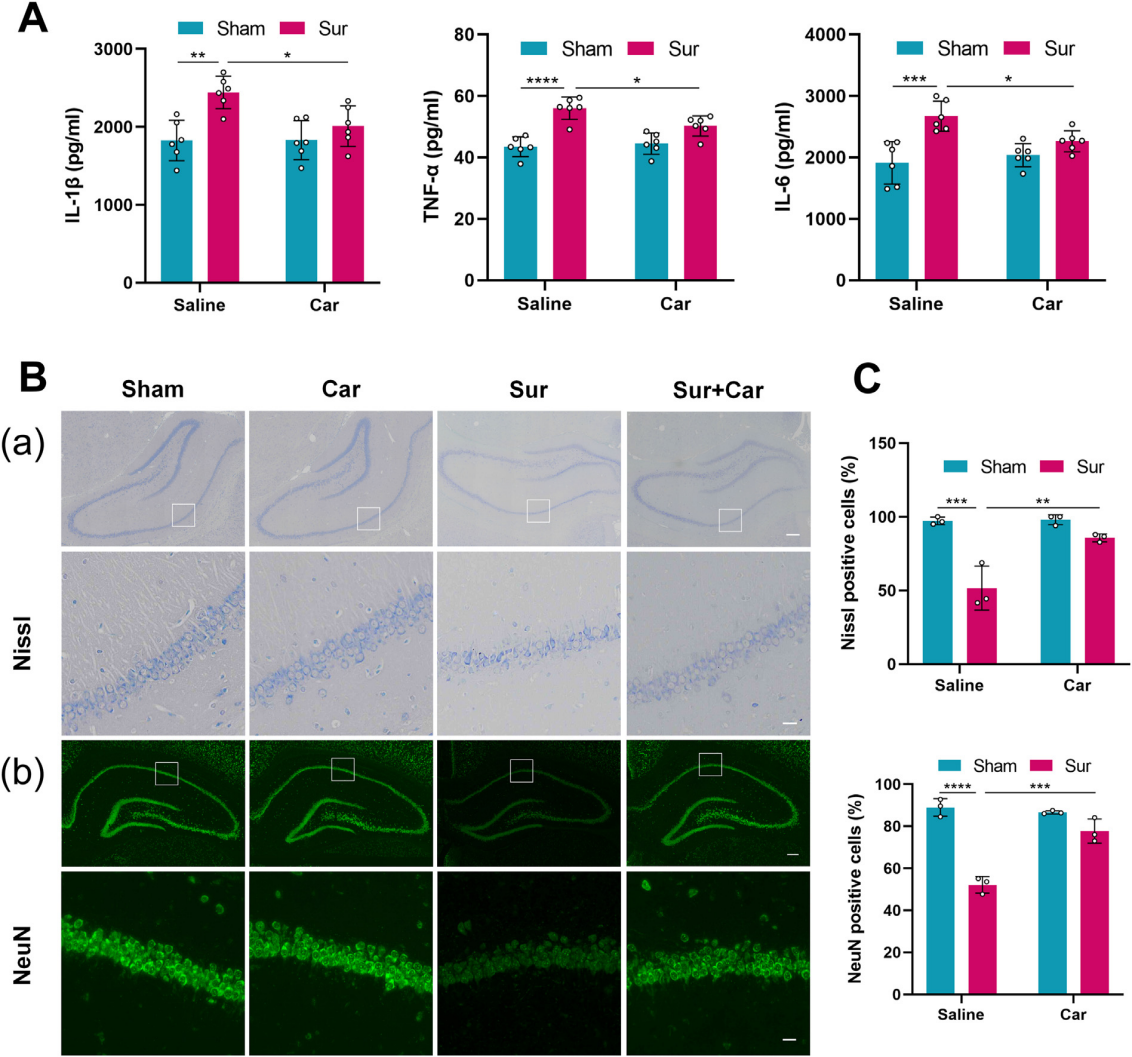
Influences of carnosine on central inflammation and neuronal injury induced by surgery in the hippocampus of aged rats. **A** ELISA was used to detect the expression levels of IL-1β (Sham: Sur, F (1, 20) ​= ​6.141, p ​= ​0.0017; Sur: Sur ​+ ​Car, F (1, 20) ​= ​4.293, p ​= ​0.0306), TNF-α (Sham: Sur, F (1, 20) ​= ​9.035, p ​< ​0.0001; Sur: Sur ​+ ​Car, F (1, 20) ​= ​4.145, p ​= ​0.0381) and IL-6 (Sham: Sur, F (1, 20) ​= ​7.526, p ​= ​0.0002; Sur: Sur ​+ ​Car, F (1, 20) ​= ​4.043, p ​= ​0.0442). Data are expressed as mean ​± ​SD (n ​= ​6 per group). ∗p ​< ​0.05, ∗∗p ​< ​0.01, ∗∗∗p ​< ​0.001, ∗∗∗∗p ​< ​0.0001. **B(a)** Nissl staining showing morphological neuronal changes in the hippocampal CA1 subregion of aged rats 24 ​h after surgery. Scale bar ​= ​200 ​μm (upper panel). Scale bar ​= ​20 ​μm (lower panel). **B(b)** NeuN staining showing the survival of neurons in the hippocampal CA1 subregion of aged rats 24 ​h after surgery. Scale bar ​= ​200 ​μm (upper panel). Scale bar ​= ​20 ​μm (lower panel). **C** The percentage of intact neurons (Nissl+, Sham: Sur, F (1, 8) ​= ​10.05, p ​= ​0.0005; Sur: Sur ​+ ​Car, F (1, 8) ​= ​7.525, p ​= ​0.0031) and living neurons (NeuN+, Sham: Sur, F (1, 8) ​= ​15.63, p ​< ​0.0001; Sur: Sur ​+ ​Car, F (1, 8) ​= ​10.87, p ​= ​0.0003) in the hippocampal CA1 subregion. Data are expressed as mean ​± ​SD (n ​= ​3 per group). ∗∗p ​< ​0.01, ∗∗∗p ​< ​0.001, ∗∗∗∗p ​< ​0.0001.

The hippocampus plays a crucial role in memory and cognition [39], and POCD is thought to be related to neuroinflammation and neuronal damage in the hippocampus. We first examined the expression levels of inflammatory cytokine in the hippocampus using ELISA. The levels of IL-1β, TNF-α and IL-6 in the surgery group were significantly increased than in the sham group. Carnosine treatment significantly down-regulated the levels of IL-1β, TNF-α and IL-6 compared with the surgery group (Fig. 3 A). Further, we employed Nissl staining to observe neuronal morphological changes in the hippocampal CA1 subregion. Neurons in the sham group had intact neuronal bodies and clear Nissl bodies, whereas neurons in the surgery group had shrunken cell bodies and pyknotic nuclei (Fig. 3 B(a)). The number of Nissl-positive cells in the surgery group was observably reduced compared with the sham group, and carnosine treatment significantly increased the proportion of Nissl-positive cells (Fig. 3C). Moreover, we performed NeuN staining to detect surviving neurons in the hippocampal CA1 subregion. The proportion of NeuN-positive cells in the surgery group was markedly lower than that in the sham group, which was increased by carnosine treatment (Fig. 3 B(b), C). Taken together, these data indicated that carnosine could attenuate surgery-induced central inflammation and neuronal injury.

### Carnosine decreased surgery-induced expression of NLRP3 inflammasome in astrocytes in the hippocampus of aged rats

**Fig. 4:**
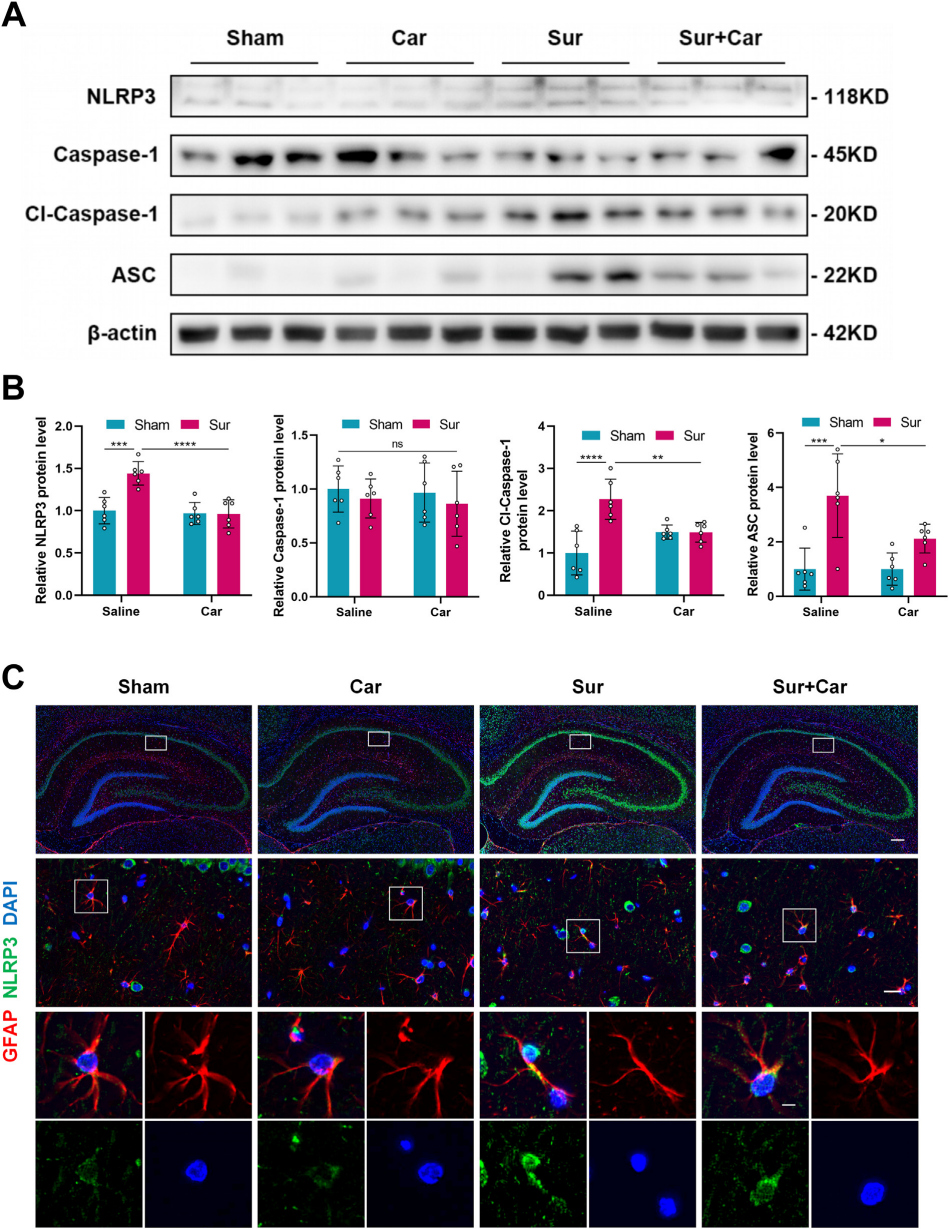
Effect of carnosine on surgery-induced expression of NLRP3 inflammasome in astrocytes in the hippocampus of aged rats. **A** Western blot analysis was used to show the protein expression levels of NLRP3, Caspase-1, Cl-Caspase-1 and ASC in the hippocampal region of aged rats 24 ​h after surgery and the bands came from three rats. **B** Statistical graphs of relative protein expression of NLRP3 (Sham: Sur, F (1, 20) ​= ​7.282, p ​= ​0.0003; Sur: Sur ​+ ​Car, F (1, 20) ​= ​7.904, p ​< ​0.0001), Caspase-1, Cl-Caspase-1 (Sham: Sur, F (1, 20) ​= ​8.193, p ​< ​0.0001; Sur: Sur ​+ ​Car, F (1, 20) ​= ​5.051, p ​= ​0.0095) and ASC (Sham: Sur, F (1, 20) ​= ​6.978, p ​= ​0.0004; Sur: Sur ​+ ​Car, F (1, 20) ​= ​4.080, p ​= ​0.0419). Data are expressed as mean ​± ​SD (n ​= ​6 per group). ∗p ​< ​0.05, ∗∗p ​< ​0.01, ∗∗∗p ​< ​0.001, ∗∗∗∗p ​< ​0.0001. **C** Immunofluorescence staining showing the localization and expression of GFAP (red) and NLRP3 (green) in the hippocampal CA1 subregion of aged rats 24 ​h after surgery. Scale bar ​= ​200 ​μm (upper panel). Scale bar ​= ​20 ​μm (middle panel). Scale bar ​= ​5 ​μm (lower panel).

Astrocytes play an essential role in neuroinflammation in the hippocampus, and NLRP3 has high expression levels in astrocytes. Using Western blot, we found that surgery led to elevated levels of NLRP3, Cl-Caspase-1 and ASC protein expression in the hippocampus of aged rats, while pretreatment with carnosine could reverse these changes. Interestingly, there was no significant difference in Caspase-1 expression among the groups (Fig. 4 A, B). GFAP is a type III intermediate filament that is a marker of mature astrocytes [40]. Consistent with the result of the western bolt, the co-localization intensity of GFAP and NLRP3 increased in the hippocampal CA1 subregion of aged rats after surgery, and carnosine administration alleviated this change (Fig. 4C). These data suggested that surgery-induced expression of NLRP3 inflammasome in astrocytes in the hippocampus of aged rats was decreased by carnosine.

### Carnosine reduced astrocytes pyroptosis induced by surgery in the hippocampus of aged rats

**Fig. 5:**
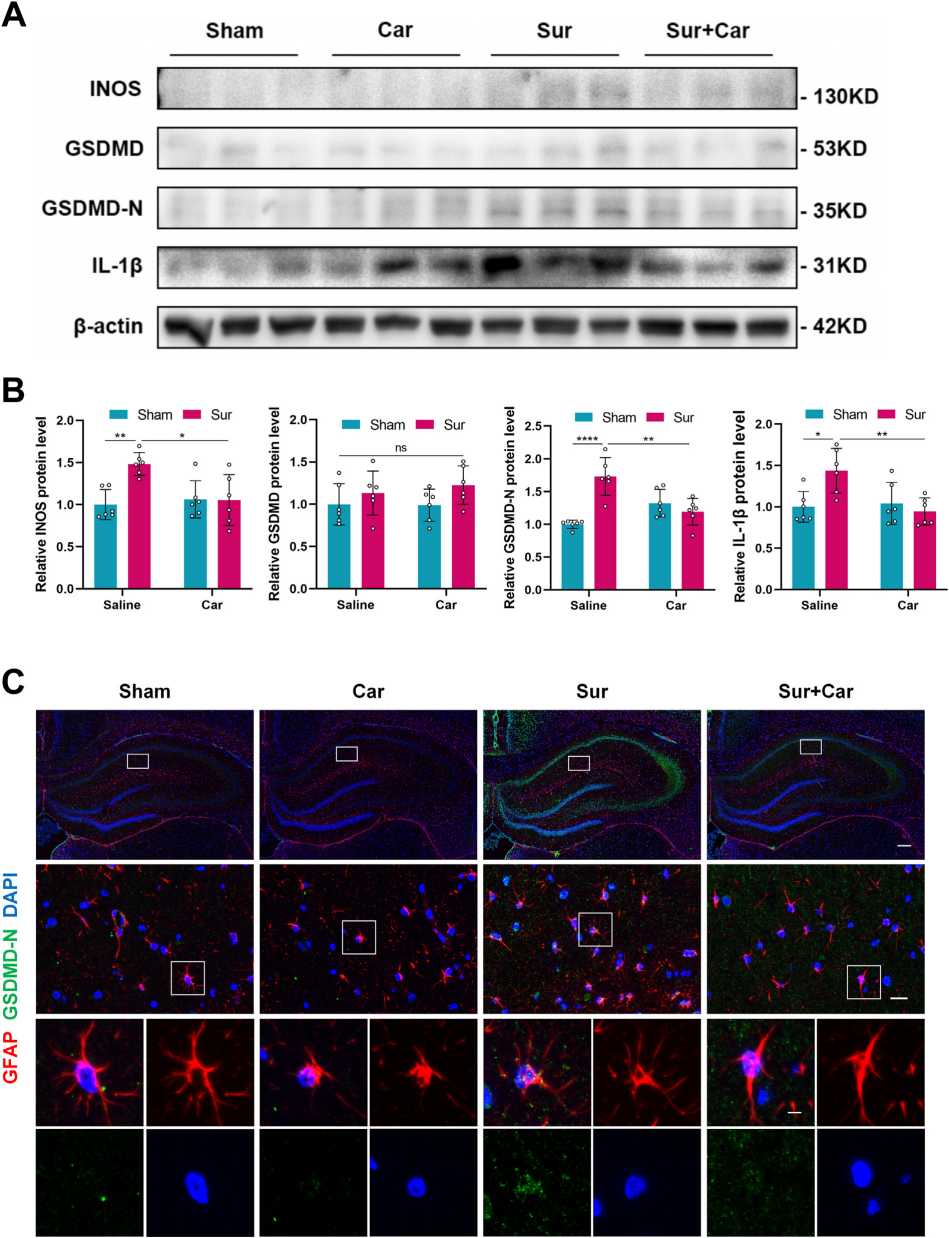
Effect of carnosine on astrocytes pyroptosis induced by surgery in the hippocampus of aged rats. **A** Western blot analysis was used to show the protein expression levels of INOS, GSDMD, GSDMD-N and IL-1β in the hippocampal region of aged rats 24 ​h after surgery and the bands came from three rats. **B** Statistical graphs of relative protein expression of INOS (Sham: Sur, F (1, 20) ​= ​5.430, p ​= ​0.0052; Sur: Sur ​+ ​Car, F (1, 20) ​= ​4.821, p ​= ​0.0136), GSDMD, GSDMD-N (Sham: Sur, F (1, 20) ​= ​8.632, p ​< ​0.0001; Sur: Sur ​+ ​Car, F (1, 20) ​= ​6.375, p ​= ​0.0011) and IL-1β (Sham: Sur, F (1, 20) ​= ​4.796, p ​= ​0.0142; Sur: Sur ​+ ​Car, F (1, 20) ​= ​5.413, p ​= ​0.0053). Data are expressed as mean ​± ​SD (n ​= ​6 per group). ∗p ​< ​0.05, ∗∗p ​< ​0.01, ∗∗∗∗p ​< ​0.0001. **C** Immunofluorescence staining showing the localization and expression of GFAP (red) and GSDMD-N (green) in the hippocampal CA1 subregion of aged rats 24 ​h after surgery. Scale bar ​= ​200 ​μm (upper panel). Scale bar ​= ​20 ​μm (middle panel). Scale bar ​= ​5 ​μm (lower panel).

The assembled NLRP3 inflammasome can activate caspase-1, induce GSDMD-dependent pyroptosis and promote the release of IL-1β [41]. And INOS is a typical proinflammatory mediator. We further examined astrocytic pyroptosis executed by the activation of NLRP3 inflammasome and the release of inflammatory cytokines. Western blot results showed that carnosine treatment markedly reduced the protein expressions of INOS, GSDMD-N and IL-1β in the hippocampus of aged rats 24 ​h after the operation. However, there was no significant difference in the expression of GSDMD protein among the four groups (Fig. 5 A, B). Meanwhile, double-antigen immunofluorescence staining of GFAP and GSDMD-N demonstrated that surgery elevated the expression of GFAP and GSDMD-N co-localization in the hippocampal CA1 subregion of aged rats, whereas carnosine treatment significantly decreased this expression (Fig. 5C). Together, these results suggested that carnosine ameliorated surgery-induced astrocytic pyroptosis in the hippocampus of aged rats and reduced the production of inflammatory mediators.

### Carnosine reduced the activation of the NLRP3 inflammasome in LPS-induced primary astrocytes

**Fig. 6:**
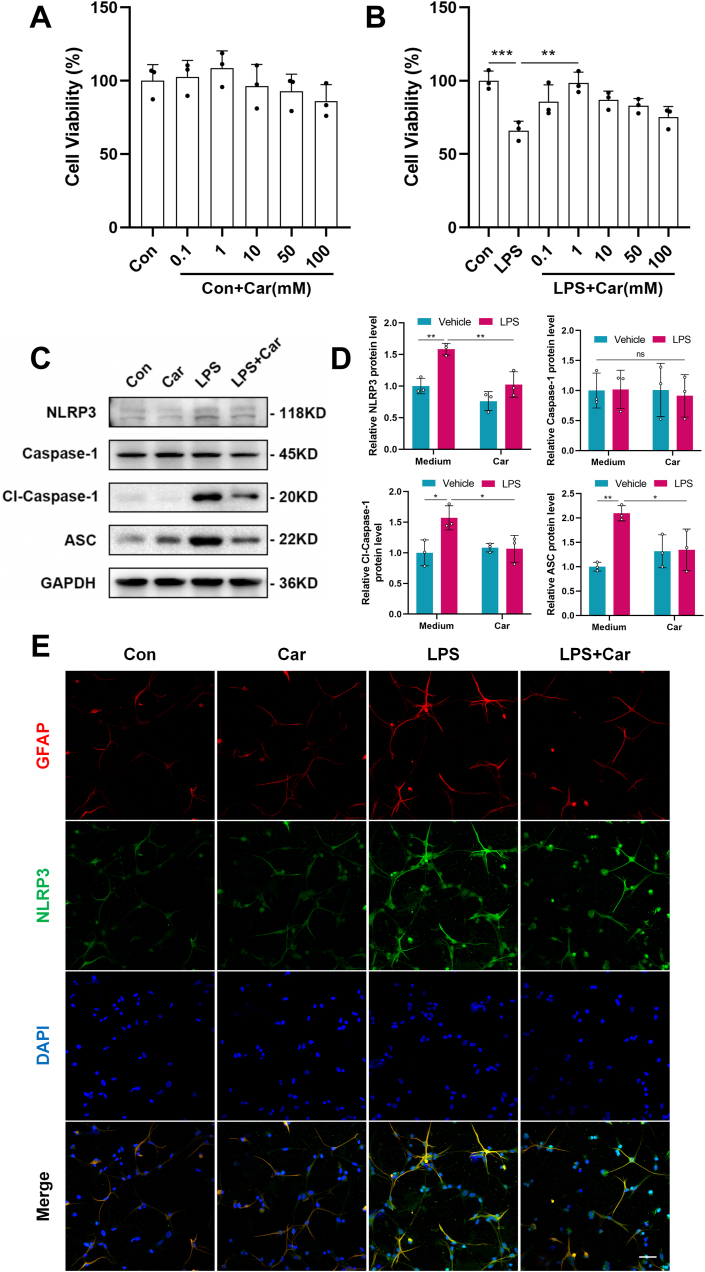
Effect of carnosine on the activation of the NLRP3 inflammasome in LPS-induced primary astrocytes. **A** CCK-8 assays were used to determine the effect of carnosine on the survival rate of primary astrocytes. Primary astrocytes were treated with different concentrations of carnosine (0.1, 1, 10, 50 and 100 ​mM) for 6 ​h. **B** CCK-8 assays were used to determine the effect of carnosine on the viability of primary astrocytes treated with LPS. Primary astrocytes were pretreated with different concentrations of carnosine (0.1, 1, 10, 50 and 100 ​mM) for 0.5 ​h, then treated with LPS (100 ​ng/ml) for 6 ​h. Primary astrocytes were pretreated with carnosine (1 ​mM) for 0.5 ​h, followed by LPS (100 ​ng/ml) stimulation for 6 ​h (Con: LPS, F (1, 14) ​= ​7.974, p ​= ​0.0009; LPS: LPS ​+ ​Car1, F (1, 14) ​= ​7.610, p ​= ​0.0015). **C** Western blot analysis was used to determine the protein expression levels of NLRP3, Caspase-1, Cl-Caspase-1 and ASC. **D** Statistical graphs of relative protein expression of NLRP3 (Con: LPS, F (1, 8) ​= ​6.871, p ​= ​0.0055; LPS: LPS ​+ ​Car, F (1, 8) ​= ​6.588, p ​= ​0.0071), Caspase-1, Cl-Caspase-1 (Con: LPS, F (1, 8) ​= ​5.341, p ​= ​0.0225; LPS: LPS ​+ ​Car, F (1, 8) ​= ​4.726, p ​= ​0.0411) and ASC (Con: LPS, F (1, 8) ​= ​6.633, p ​= ​0.0068; LPS: LPS ​+ ​Car, F (1, 8) ​= ​4.533, p ​= ​0.0498). **E** Immunofluorescence staining was performed for GFAP (red) and NLRP3 (green) expression. Scale bar ​= ​50 ​μm. All the data are presented as mean ​± ​SD of three independent experiments. ∗p ​< ​0.05, ∗∗p ​< ​0.01, ∗∗∗p ​< ​0.001.

The protective effect of carnosine treatment in mediating NLRP3 inflammasome activation was also explored in primary astrocytes in POCD in vitro. Previous studies have demonstrated that 100 ​ng/ml LPS treatment leads to the activation of the NLRP3 inflammasome in astrocytes [20,37], so we chose this concentration for the experiment. To assess the optimal concentration of carnosine, we performed CCK-8 assays to monitor cell viability. A carnosine concentration less than or equal to 100 ​mM had little impact on the cell viability of primary astrocytes (Fig. 6 A). A carnosine concentration of 1 ​mM had the greatest effect on restoring the cell viability of LPS-induced primary astrocytes (Fig. 6 B). So we chose the carnosine concentration of 1 ​mM for follow-up experiments. Primary astrocytes were pretreated with carnosine (1 ​mM) for 0.5 ​h, followed by LPS (100 ​ng/ml) stimulation for 6 ​h. Western blot results showed that carnosine pretreatment significantly reduced the expression NLRP3, Cl-Caspase-1 and ASC in primary astrocytes compared with LPS alone, while there was no difference in Caspase-1 expression among the groups (Fig. 6C, D). Furthermore, we performed double-antigen immunofluorescence staining using astroglial cell-specific GFAP and NLRP3. The expression of GFAP and NLRP3 co-localization was evident in primary astrocytes after exposure to LPS, whereas carnosine pretreatment alleviated this phenomenon (Fig. 6 E). Taken together, these findings indicated that carnosine could reduce the activation of the NLRP3 inflammasome in LPS-induced primary astrocytes.

### Carnosine alleviated LPS-induced pyroptosis and inflammation in primary astrocytes

**Fig. 7:**
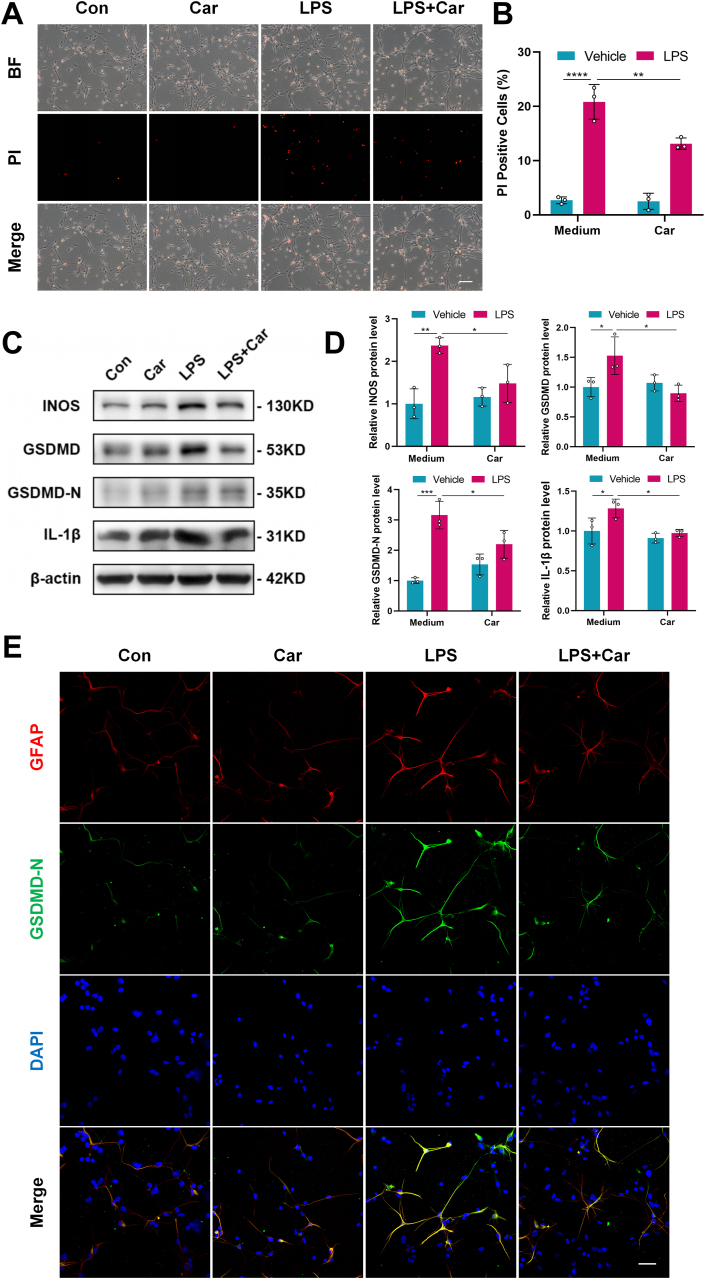
Influences of Carnosine on LPS-induced pyroptosis and inflammation in primary astrocytes. Primary astrocytes were pretreated with carnosine (1 ​mM) for 0.5 ​h, followed by LPS (100 ​ng/ml) stimulation for 6 ​h. **A** Propidium iodide (PI) staining was used to find PI-positive cells (pyroptotic cell death). Scale bar ​= ​100 ​μm. **B** Statistical graph of PI-positive cells (Con: LPS, F (1, 8) ​= ​16.88, p ​< ​0.0001; LPS: LPS ​+ ​Car, F (1, 8) ​= ​7.165, p ​= ​0.0043). **C** Western blot analysis was used to determine the protein expression levels of INOS, GSDMD, GSDMD-N and IL-1β. **D** Statistical graphs of relative protein expression of INOS (Con: LPS, F (1, 8) ​= ​7.457, p ​= ​0.0033; LPS: LPS ​+ ​Car, F (1, 8) ​= ​4.839, p ​= ​0.0368), GSDMD (Con: LPS, F (1, 8) ​= ​4.529, p ​= ​0.0500; LPS: LPS ​+ ​Car, F (1, 8) ​= ​5.410, p ​= ​0.0211), GSDMD-N (Con: LPS, F (1, 8) ​= ​10.17, p ​= ​0.0004; LPS: LPS ​+ ​Car, F (1, 8) ​= ​4.529, p ​= ​0.0500) and IL-1β (Con: LPS, F (1, 8) ​= ​4.542, p ​= ​0.0493; LPS: LPS ​+ ​Car, F (1, 8) ​= ​4.950, p ​= ​0.0330). **E** Immunofluorescence staining was performed for GFAP (red) and GSDMD-N (green) expression. Scale bar ​= ​50 ​μm. All the data are presented as mean ​± ​SD of three independent experiments. ∗p ​< ​0.05, ∗∗p ​< ​0.01, ∗∗∗p ​< ​0.001, ∗∗∗∗p ​< ​0.0001.

Next, we investigated the effect of carnosine on the pyroptosis and inflammatory response of primary astrocytes induced by LPS. We used PI staining to evaluate pyroptotic cell death. As expected, the number of PI-positive cells in the LPS group significantly increased compared with the control group. Carnosine pretreatment markedly reduced the number of PI uptake cells compared with the LPS group suggesting that carnosine could inhibit LPS-induced pyroptosis of primary astrocytes (Fig. 7 A, B). Moreover, carnosine pretreatment significantly inhibited the protein expression of INOS, GSDMD, GSDMD-N and IL-1β in LPS-activated primary astrocytes (Fig. 7C, D). To verify these results, we performed double-antigen immunofluorescence staining to assess the expression of GFAP and GSDMD-N co-localization. Compared with the control group, the co-expression of GFAP and GSDMD-N in primary astrocytes was strongly enhanced in the LPS group, while pretreatment with carnosine inhibited this effect (Fig. 7 E). Taken together, these results demonstrated that carnosine alleviated LPS-induced pyroptosis and inflammation in primary astrocytes.

## Discussion

**Fig. 8:**
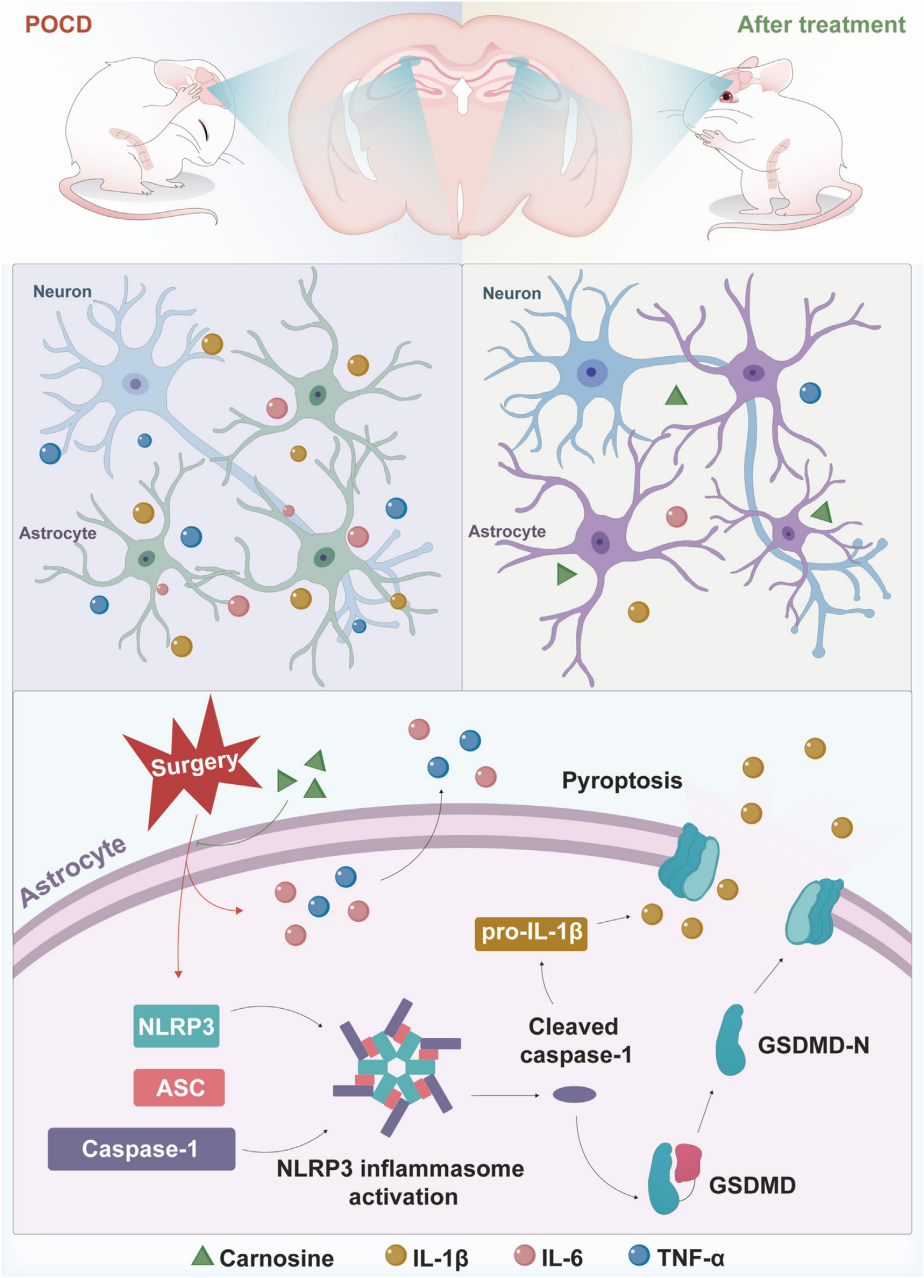
Schematic illustration of the mechanism underlying carnosine improves postoperative cognitive dysfunction.

In the present study, we revealed the role of carnosine in improving postoperative cognition and its potential mechanisms (Fig. 8). Current studies indicated that carnosine reduced the activation of NLRP3 inflammasome and subsequent pyroptosis in astrocytes both in vivo and in vitro. Then, carnosine attenuated central inflammation and neuronal damage in the hippocampus and ameliorated learning and memory impairment caused by surgery.

With the extension of life expectancy and the improvement of medical levels in today's society, more and more people choose surgery to fight diseases, and the importance of postoperative complications such as POCD has been increasingly recognized. POCD is a common postoperative disorder that affects learning, memory and cognitive function, occurring in about 10–54% of individuals within the first few weeks following surgery [42]. The incidence of POCD is particularly prominent in the elderly, and advanced age is considered to be an important risk factor for POCD [43,44]. Therefore, in this study, aged rats were selected as research objects and exploratory laparotomy was performed to establish the POCD model.

Carnosine is a natural endogenous molecule containing histidine, which has an anti-inflammatory and neuroprotective effect [25,45]. Previous research has shown that carnosine prevents serotonin-derived melanoid (SDM) synthesis and neuronal impairment caused by sevoflurane-induced sequestration of age-related acrolein [46]. In addition, few studies have investigated the prevention or treatment of carnosine on POCD. We evaluated the effect of carnosine on surgery-induced cognitive impairment behavior. After using OFT to rule out the effect of surgery on the mobility of rats, we found that carnosine was effective in preventing postoperative cognitive deficits in rats by Y-maze and MWM tests, proving the role of carnosine on POCD.

The hippocampus is a brain region that plays a key role in learning and memory consolidation as well as affective behavior and emotion regulation [47]. A study has shown that hippocampal-dependent learning and memory are particularly vulnerable to surgery in aged animals [48]. So we focused on the hippocampus in rats. The systemic inflammatory response caused by surgery increases the level of inflammatory factors in plasma, and peripheral inflammatory factors infiltrate into the CNS to induce neuroinflammation, and then the formation of hippocampal neurons and the plasticity of hippocampal morphology associated with learning and memory are inhibited, eventually leading to impaired cognitive function [49].

As we know, nitric oxide is produced by a group of enzymes called nitric oxide synthase (NOS), INOS is expressed in immune stimulation or inflammation and has severe neurotoxic effects [50]. Meanwhile, IL-1β is an inducible cytokine that can be produced by astrocytes and has been shown to play a central role in mediating neuroinflammation in most CNS-related diseases. It can also activate astrocytes, leading to the downstream synthesis of other pro-inflammatory and chemotactic mediators in the CNS and damaging the hippocampal nerve [51,52]. Moreover, TNF-α and IL-6 have been considered crucial proinflammatory cytokines and play an important role in the onset and progression of neuroinflammation-related diseases [53]. Here, we observed an increase in the expression of INOS protein and the release of IL-1β, TNF-α and IL-6 in the hippocampus of aged rats induced by surgery, which were all reduced by carnosine intervention indicating that carnosine could improve surgery-caused central inflammation.

Neuroinflammation can exacerbate neuronal damage in the hippocampus and affect hippocampal neurogenesis, leading to many neurocognitive diseases [54,55]. As expected, in this study, we found that carnosine treatment significantly improved neurological deficits and increased neuronal survival in the CA1 region after surgery. This is similar to the findings of a new study investigating the effects of intragastric-administered carnosine in a rat model of type 2 diabetes mellitus [56].

Astrocytes and neurons are thought to be the main users of carnosine in the brain [57]. Astrocytes have powerful pro-inflammatory potential and are a critical regulator of CNS inflammation [58,59]. In addition, NLRP3 has high expression levels in astrocytes. The NLRP3 inflammasome is a multimeric protein complex that is an important component of the innate immune system. It is composed of three main components: a pattern domain NLRP3, a central adaptor recognition ASC and an inflammatory caspase-1 [60]. NLRP3 inflammasome activation and dysregulation are also considered to be an important process in neuroinflammation, which contributes to the pathogenesis of a variety of cognitive disorders such as AD, PD, and Huntington's disease (HD) [61]. Meanwhile, accumulating evidence has shown that the overactivation of NLRP3 inflammasome promotes the development of POCD [62]. Therefore, we speculated that the activation of NLRP3 inflammasome in astrocytes was a basis for neuroinflammation and cognitive impairment. In the present study, we observed that aged rats that underwent exploratory laparotomy displayed increased protein expression of NLRP3, Cl-Caspase-1 and ASC in the hippocampal astrocytes. Consistent with the in vivo results, NLRP3, Cl-Caspase-1 and ASC protein expression were largely elevated in LPS-stimulated primary astrocytes, suggesting that the activation of NLRP3 inflammasome in astrocytes did play a critical role in the progression of POCD. Carnosine pretreatment significantly reduced the activation of NLRP3 inflammasome in astrocytes in both in vivo and in vitro models.

Pyroptosis is a proinflammatory type of cell death initiated by the activation of inflammasome [63]. NLRP3 assembles a multiprotein platform resulting in caspase-1 activation, then activated caspase-1 cleaves GSDMD to release the GSDMD-N which induces pyroptosis, it also cleaves pro-cytokines including pro-IL-1β into their active forms which are released through GSDMD pores [64,65]. In the current study, we found that surgery significantly increased GSDMD-N and IL-1β expression in the hippocampus of aged rats. These findings were further confirmed in vitro experiments of primary astrocytes stimulated by LPS. Taken together, our results inferred that surgery could activate NLRP3 inflammasome-mediated pyroptosis in astrocytes, which is alleviated with carnosine administration. In another study, researchers also found that carnosine reduces podocyte injury by inhibiting pyroptosis via the targeting of caspase-1 [66].

Of note, our research is not without limitations. First, we only explored the effect of carnosine on NLRP3 inflammasome-mediated pyroptosis in astrocytes since astrocytes are the main users of carnosine in the brain. At present, it has been found that microglia pyroptosis plays a certain role in cognitive impairment induced by surgery and anesthesia [67,68], so the role of carnosine may not be limited to astrocytes and needs further study. Second, carnosine alleviated neuroinflammation and neuronal damage in the hippocampus, we could not rule out the possibility that other brain regions may underlie the functions of carnosine. Third, our experiment focused on short-term cognitive function after the operation, but the long-term effects are still unknown.

In summary, our study demonstrates that carnosine could ameliorate POCD of aged rats via inhibiting NLRP3-mediated astrocytes pyroptosis and neuroinflammation. These results also highlight the neuroprotective effect of carnosine is a promising therapeutic target for cognitive impairment in POCD (Supplementary Fig. 1, Supplementary Fig. 2).

## Ethics approval and consent to participate

All experimental procedures were conducted in accordance with the Ethics Committee of Zhejiang Chinese Medicine University.

## Funding

This project was sponsored by the 10.13039/501100001809National Natural Science Foundation of China (No. 81901087; 82271445), the 10.13039/501100004731Natural Science Foundation of Zhejiang Province (No. LY21H090005), the Construction Fund of Key Medical Disciplines of Hangzhou (Anesthesia and Pain Medicine OO20200484), and the Medical and Health Science and Technology Project of Zhejiang Provincial (Grant: 2021KY868).

## Authors’ contributions

Jiahong Shen, Jiawen Xu and Yuxin Wen contributed equally to this research. Jiahong Shen, Jiawen Xu and Jianliang Sun designed the experiments. Jiahong Shen and Yuxin Wen performed the experiments and analyzed the data. Zili Tang and Jiaqi Li assisted with the experiments. Jiahong Shen wrote the manuscript. Jiawen Xu and Jianliang Sun revised the manuscript. All authors read and approved the final manuscript.

## Consent for publication

Not applicable.

## Availability of Data and materials

The datasets used and/or analyzed during the current study are available from the corresponding author upon reasonable request.

## Declaration of competing interest

The authors declare that they have no known competing financial interests or personal relationships that could have appeared to influence the work reported in this paper.
