# Supplementary material for: Carnosine ameliorates postoperative cognitive dysfunction of aged rats by limiting astrocytes pyroptosis
Source: Neurotherapeutics. 2024 Apr 25;21(4):e00359. doi: 10.1016/j.neurot.2024.e00359 (PMC11301240; doi:10.1016/j.neurot.2024.e00359)
Supplement: Multimedia component 1 [file mmc1.docx]

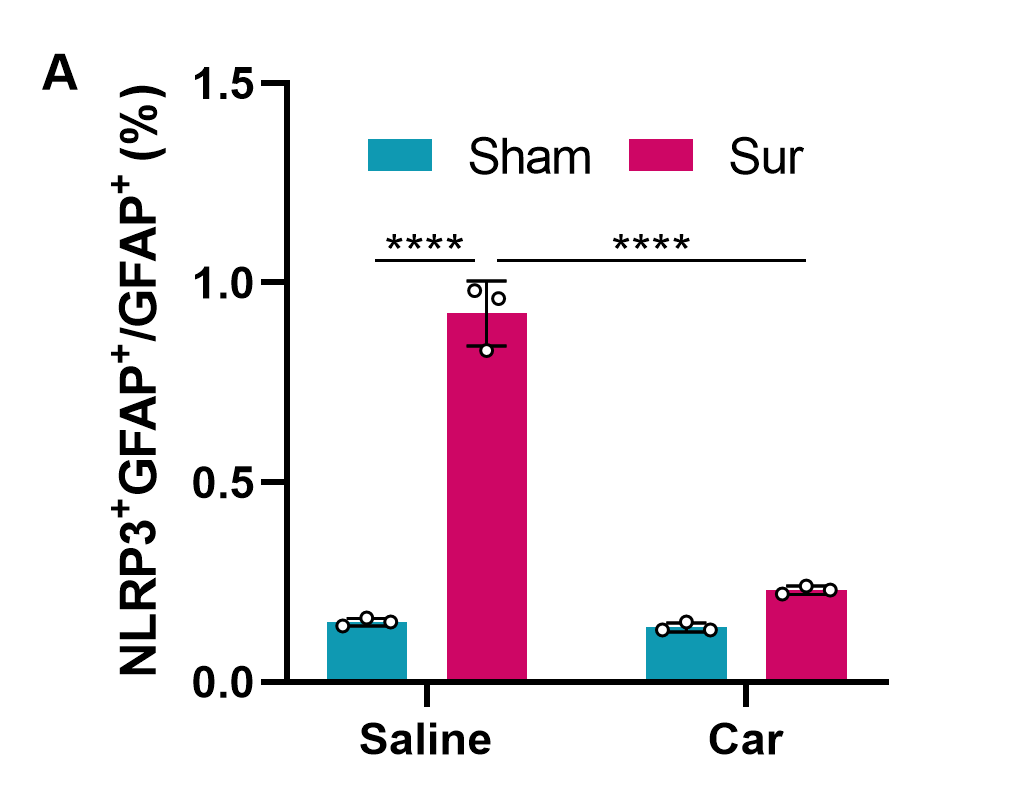

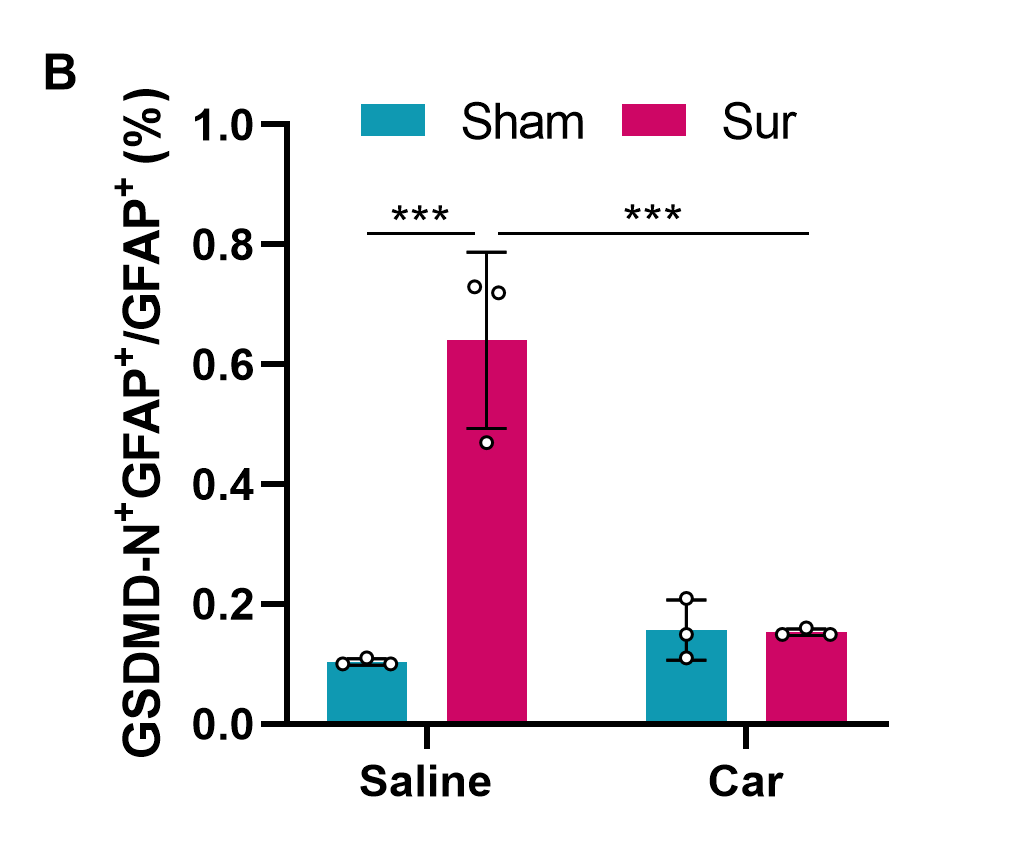


**Supplementary Fig.1 A** Quantification of the IF staining of GFAP and NLRP3 in the hippocampal CA1 subregion of aged rats 24 h after surgery (Sham: Sur, F (1, 8) = 32.10, p < 0.0001; Sur: Sur+Car, F (1, 8) = 28.78, p < 0.0001). **B** Quantification of the IF staining of GFAP and GSDMD-N in the hippocampal CA1 subregion of aged rats 24 h after surgery (Sham: Sur, F (1, 8) = 11.93, p = 0.0001; Sur: Sur+Car, F (1, 8) = 10.81, p = 0.0003). Data are expressed as mean ± SD (n = 3 per group). ***p < 0.001, ****p < 0.0001.


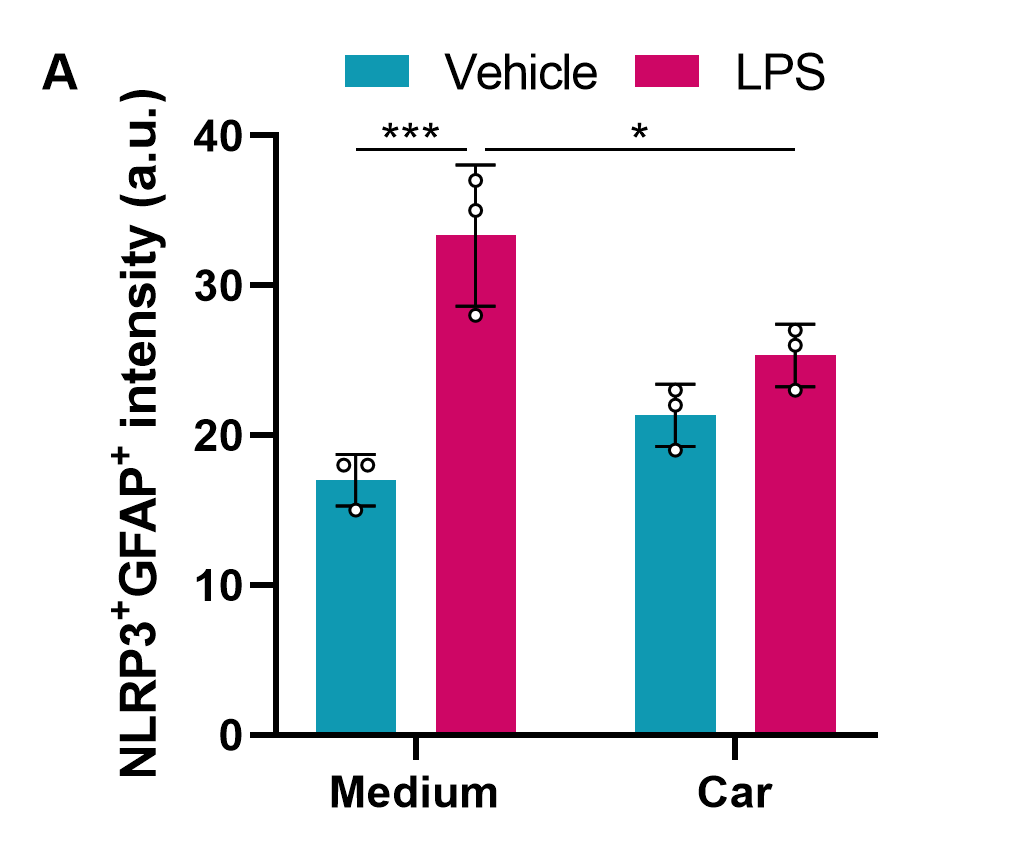

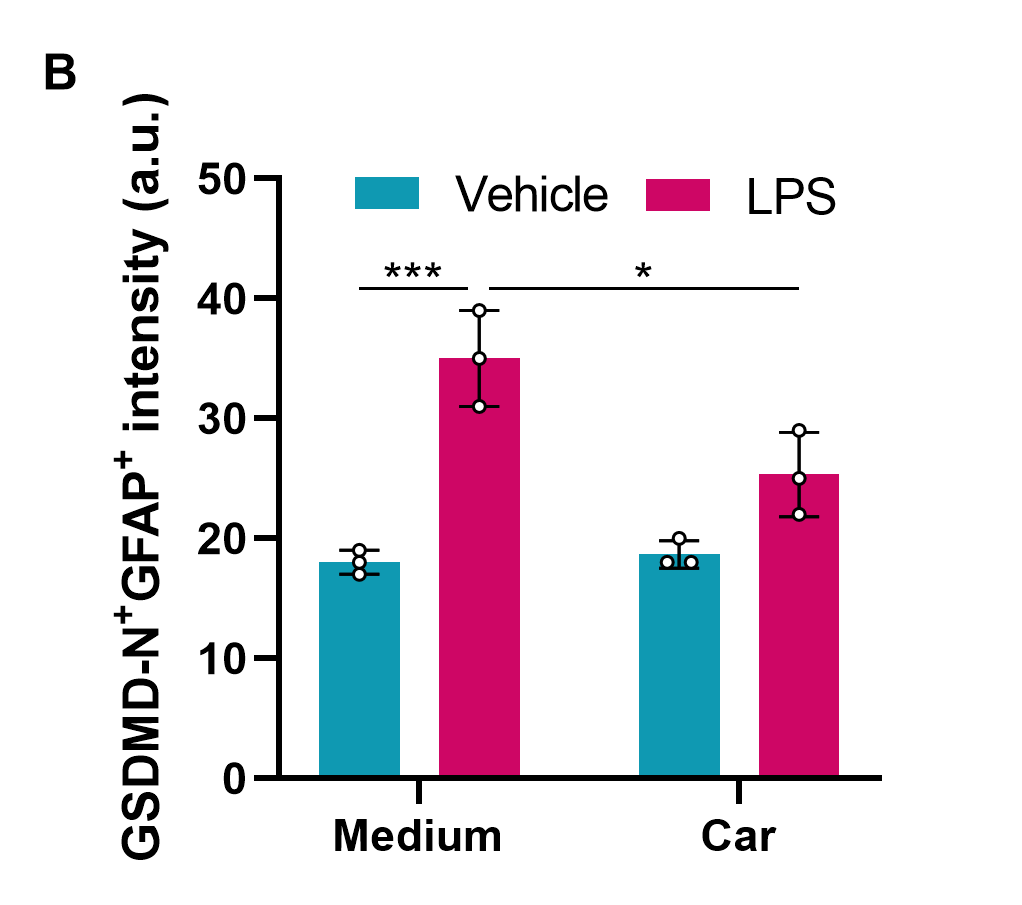


**Supplementary Fig.2 A** Quantification of the IF staining of GFAP and NLRP3 in LPS-induced primary astrocytes (Con: LPS, F (1, 8) = 9.703, p = 0.0006; LPS: LPS+Car, F (1, 8) = 4.753, p = 0.0400). **B** Quantification of the IF staining of GFAP and GSDMD-N in LPS-induced primary astrocytes (Con: LPS, F (1, 8) = 10.63, p = 0.0003; LPS: LPS+Car, F (1, 8) = 6.047, p = 0.0116). All the data are presented as mean ± SD of three independent experiments. *p < 0.05, ***p < 0.001.
